# Maternal depression or anxiety during pregnancy and offspring type 1 diabetes: a population-based family-design cohort study

**DOI:** 10.1136/bmjdrc-2023-003303

**Published:** 2023-04-20

**Authors:** Awad I Smew, Cecilia Lundholm, Tong Gong, Lars Sävendahl, Paul Lichtenstein, Bronwyn K Brew, Catarina Almqvist

**Affiliations:** 1 Department of Medical Epidemiology and Biostatistics, Karolinska Institutet, Stockholm, Sweden; 2 Department of Women’s and Children’s Health, Karolinska Institutet, Stockholm, Sweden; 3 Pediatric Endocrinology Unit, Astrid Lindgren Children’s Hospital, Karolinska University Hospital, Stockholm, Sweden; 4 National Perinatal Epidemiology and Statistics Unit, Centre for Big Data Research in Health and School of Clinical Medicine, University of New South Wales, Sydney, New South Wales, Australia; 5 Pediatric Allergy and Pulmonology Unit, Astrid Lindgren Children’s Hospital, Karolinska University Hospital, Stockholm, Sweden

**Keywords:** pregnancy, epidemiology, diabetes mellitus, type 1, stress, psychological

## Abstract

**Introduction:**

To investigate the association between maternal depression/anxiety during pregnancy and offspring type 1 diabetes, to assess the specific importance of exposure during pregnancy by comparing across different exposure periods before and/or after pregnancy, and to explore potential unmeasured familial confounding.

**Research design and methods:**

This was a population-based cohort including 1 807 809 offspring born in Sweden 2002–2019. From national registers, data were available on diagnosis or medication prescription for depression/anxiety in and around pregnancy, as well as incident cases of type 1 diabetes defined through diagnosis or insulin treatment. Associations were examined using flexible parametric and Cox regression models. Familial confounding was explored using paternal exposure as a negative control and by comparing offspring exposed to maternal depression/anxiety with their unexposed siblings.

**Results:**

For exposure during pregnancy, maternal depression/anxiety was associated with an increased risk of offspring type 1 diabetes onset after, but not before, 8 years of age (adjusted HR (aHR) 1.21 (95% CI 1.03 to 1.42]). Exposure occurring only during pregnancy was similarly associated to type 1 diabetes (aHR 1.24 (0.96 to 1.60)), whereas exposure occurring only before pregnancy was not (aHR 0.91 (0.64 to 1.30)). Associations were close to the null for paternal depression/anxiety (aHR 0.95 (0.72 to 1.25)), and point estimates were above 1 in sibling comparisons, although with wide CIs (aHR 1.36 (0.82 to 2.26)).

**Conclusions:**

Maternal depression/anxiety specifically during pregnancy seems to be associated with offspring type 1 diabetes. Paternal negative control and sibling comparisons indicate that the results cannot entirely be explained by familial confounding.

**WHAT IS ALREADY KNOWN ON THIS TOPIC**

- Previous research has focused on childhood stress as a trigger for type 1 diabetes but less is known on potential fetal programming by maternal stress during pregnancy. Depression/anxiety as a proxy of stress, specifically during pregnancy compared with before or after pregnancy, has not previously been studied in relation to offspring type 1 diabetes risk. Furthermore, it is not clear if associations found are due to residual confounding by familial factors.

**WHAT THIS STUDY ADDS**

- This study demonstrates that offspring to mothers who experienced depression/anxiety specifically during pregnancy had an increased risk of type 1 diabetes and that the associations found are not entirely explained by familial confounding.

**HOW THIS STUDY MIGHT AFFECT RESEARCH, PRACTICE OR POLICY**

- These findings contribute to identifying maternal stress during pregnancy as a risk factor for offspring type 1 diabetes and highlight the importance for future research in understanding the pathways through which early-life risk factors impact disease initiation and progression.

## Introduction

Type 1 diabetes is one of the most common chronic autoimmune disorders in children with peaks in onset between 5 and 7 years of age and around or during puberty.^1^ The incidence has increased worldwide over past decades, with the highest rates in Scandinavia (30–60 cases per 100 000).^2^ Searching for factors explaining this rise is a targeted area of research to identify potentially modifiable predictors, with many studies pointing to the importance of environmental determinants.^3^ A range of factors such as rapid weight gain, viral infections, diet and childhood psychological distress are thought to play a role in triggering the development of overt disease from a subclinical prodromal state of circulating islet autoantibodies, particularly in children with a genetic predisposition.^4–6^

While research has mainly focused on childhood exposures affecting autoimmunity or disease progression, less is known about fetal programming of type 1 diabetes susceptibility through maternal and perinatal factors.^5^ Studying associations between exposures during pregnancy and offspring health outcomes, also known as the Developmental Origins of Health and Disease framework,^7^ is particularly relevant for research on the etiology of type 1 diabetes since disease processes such as the first appearance of autoantibodies often begin already in the first years of life.^8^ Although some risk factors such as higher maternal age at delivery, pregestational or early gestational obesity and increased birth weight have been implicated,^9^ the body of literature examining the role of maternal stress during pregnancy in type 1 diabetes is scarce, with inconclusive results.^10–13^

Several forms of stress exist, but studying depression/anxiety as a proxy has clinical relevance given that these disorders affect many women during or around pregnancy with a prevalence of 15%–20%.^14^ Advantageously, information on this type of stress exposure can be found in routinely and prospectively collected Swedish healthcare data. Recent examples using these data include one study that found an increased risk of type 1 diabetes in children of parents diagnosed with depression, anxiety, or stress-related disorders,^15^ and another study that presented a familial coaggregation of these psychiatric diagnoses with type 1 diabetes.^16^ Yet, to the best of our knowledge, depression/anxiety specifically during pregnancy has hitherto not been examined in relation to offspring type 1 diabetes.

Furthermore, previous research in this field has not focused on understanding the timing of exposures around pregnancy. Comparing periods before, during and/or after pregnancy may help to elucidate the role of intrauterine exposure compared with time-stable factors before, or alternative exposures after pregnancy. Also, associations in observational data often suffer from residual confounding despite adjustment for measured confounders. By using family-designs drawing on known genetic and environmental sharing between different relatives (such as fathers or siblings), it is also possible to explore the role of unmeasured familial confounding and address causal relationships.^17 18^

The aims of this study were to investigate the association between maternal depression/anxiety during pregnancy and offspring type 1 diabetes in addition to assessing the specific importance of exposure during pregnancy by comparing across different exposure periods before and/or after pregnancy and to explore potential unmeasured familial confounding.

## Methods

### Study design—population and data sources

This was a nationwide cohort study of all children born between January 1, 2002 and December 31, 2019 identified from the Medical Birth Register (MBR), covering 96%–98% of births in Sweden.^19^ Thanks to the unique personal identification number given to all Swedish residents,^20^ individuals were unambiguously linked to multiple national sociodemographic and healthcare data sources (online supplemental methods). Exclusion criteria included multiparous births, mothers’ migration during pregnancy, any of offspring migration/death/type 1 diabetes onset before 1 year of age (in order to assess exposure up to 1 year post-delivery and to avoid inclusion of cases of neonatal diabetes) and missing identity of children’s parents (online supplemental figure S1).

10.1136/bmjdrc-2023-003303.supp1 Supplementary data

10.1136/bmjdrc-2023-003303.supp2 Supplementary data

### Measures

#### Outcome definition

The offspring outcome was defined as either: any diagnosis of type 1 diabetes (International Classification of Diseases Tenth Revision (ICD-10) E10) registered in the National Patient Register (NPR) or dispensation of insulin prescription (Anatomical Therapeutic Chemical classification (ATC) A10A) in the Prescribed Drug Register (PDR), both before 18 years of age. Date of disease onset was the date of diagnosis of type 1 diabetes, or if missing, the date of the first insulin prescription.^21 22^

#### Exposure definition

Maternal or paternal depression/anxiety was defined as any diagnosis for mood-related or anxiety-related disorders (ICD-10 F30-34, F38, F39) recorded in the NPR, or anxiolytic or antidepressant medication (ATC N05B, N06A). Medication was determined from either of two sources: the MBR for maternal self-reported use registered by midwives during the first antenatal care visit around gestational weeks 10–12 (available for the whole study period) and the PDR for all prescriptions of dispensed drugs for both mother and father (registered from July 1, 2005).^23^ Information from the MBR has been shown to correspond well with recorded dispensation in the PDR, particularly for antidepressant medication.^24^

**Figure 1:**
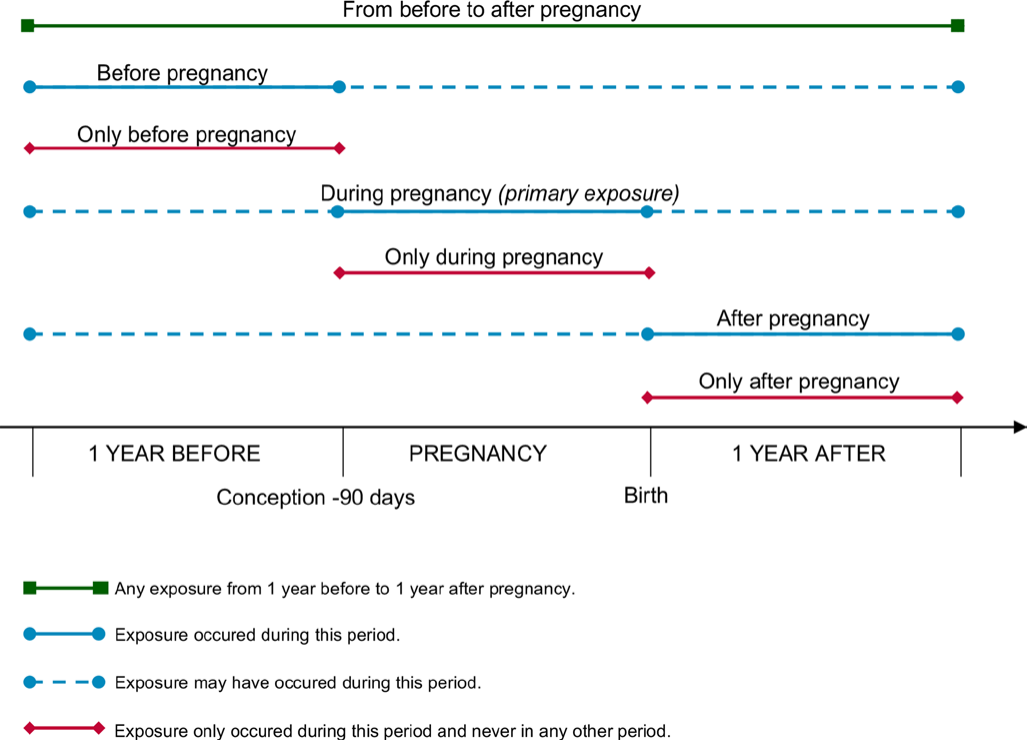
Overview of various exposure periods before, during, and/or after pregnancy. The primary exposure is maternal depression/anxiety during pregnancy. All other exposure periods are secondary.

Figure 1 displays the seven different time periods in which exposure was assessed. The primary exposure period was *during pregnancy*, defined as 90 days before conception up until delivery. Conception was calculated by subtracting the gestational age from the child’s date of birth. A secondary exposure period *from before to after pregnancy* also included time before (1 year before pregnancy period) and after pregnancy (1 year after delivery). In order to assess exposure longitudinally over the entire period and to attempt to understand if all periods contribute equally or if one period is more important than the other,^25^ exposure was additionally categorized into secondary periods where exposure occurred during the named period but may also have occurred during other periods (*before/after pregnancy*) and mutually exclusive periods where exposure only occurred in that named period and never in any other period (*only before/only during/only after pregnancy*).

#### Covariates

Potential confounders were identified using directed acyclic graphs based on literature review of associations between covariates and exposure/outcome and subject-matter knowledge.^26^ These included the maternal factors body mass index (BMI) in early pregnancy, parity, age at delivery, type 1 diabetes, and highest level of educational attainment (online supplemental methods and online supplemental figure S2).

10.1136/bmjdrc-2023-003303.supp3 Supplementary data

### Statistical analyses

For all associations between primary and secondary exposures and offspring type 1 diabetes we fitted flexible parametric models modelling the baseline hazard with restricted cubic splines (three degrees of freedom) and allowing for time-varying effects of the exposure and offspring sex and birth year (also three degrees of freedom), using the Stata package “stpm2”.^27^ Attained age was the underlying timescale with follow-up starting at 1 year of age, and ending on date of type 1 diabetes onset, emigration, death, or December 31, 2020 (end of study period), whichever occurred first. This type of time-to-event analysis was chosen given evidence for non-proportional hazards of the exposure, sex and birth year over time based on Schoenfeld residuals and visual examination of log-cumulative hazard curves, and in order to deal with different length of follow-up depending on birth year. Results are presented as hazard ratio (HR) curves by attained age. To aid with comparing to results from paternal negative control and sibling comparison described below, we also applied Cox regression models estimating HRs and 95% CI allowing for time-varying effects by two categories of attained age: (1–8, >8 years of age) as well as adjusted for sex and birth year by stratification. All flexible parametric and Cox models were adjusted for maternal early pregnancy BMI, parity, age at delivery, type 1 diabetes, and highest level of educational attainment. We further examined possible effect modification by maternal asthma, type 1 diabetes, or BMI by including interaction terms and testing for differences in HRs from Cox models using likelihood ratio tests (online supplemental methods).

A negative control exposure model was constructed based on exposure to paternal depression/anxiety *during pregnancy*. The assumption in applying a negative control for in utero factors is that paternal exposures *during pregnancy* in general should not have a direct effect on the unborn child.^17^ In brief, if similar associations are found in paternal models, it indicates the existence of similar confounding structures for mothers and fathers and suggests that the maternal estimates may be biased. Cox models for paternal exposure were adjusted for the following paternal covariates: age at delivery, type 1 diabetes, and level of educational attainment as well as sex and birth year by stratification. Additionally, both maternal and paternal models were mutually adjusted for the other’s exposure; maternal models were adjusted for paternal depression/anxiety and vice versa, in order to block a potential indirect pathway between paternal exposure and offspring type 1 diabetes, via maternal depression/anxiety.^23^

In a sibling comparison analysis of the offspring, we analyzed exposure *during pregnancy* and risk of type 1 diabetes among all full sibling-pairs within the cohort by matching each exposed offspring to their unexposed siblings. This type of analysis inherently adjusts for unmeasured confounders constant between siblings, that is, shared genetic and environmental factors, by comparing siblings discordant on both exposure to maternal stress during pregnancy and type 1 diabetes.^18^ The closer the estimate is to 1, the more likely that factors shared between siblings explain an association found in the whole population analysis. Cox regression models, stratified on sibling pair, were fitted in order to only compare within families by allowing for a family-specific baseline hazard. Models were adjusted for offspring birth year and sex as well as for confounders that vary between siblings (maternal BMI, parity, age) and are presented by category of attained age (1–8, >8 years of age). The sandwich estimator for robust standard errors was applied to deal with familial clustering.

#### Sensitivity analyses

Sensitivity analyses were conducted to investigate the robustness of the results of the primary exposure *during pregnancy* using Cox regression. They were performed on a restricted cohort of offspring born between July 1, 2006 and December 31, 2019, which ensured the same exposure classification over the entire follow-up with full coverage of the PDR from July 1, 2005 and allowed for evaluation of the risk of bias due to left censoring of the exposure in the PDR and cohort effects. First, to address potential exposure misclassification of the register-based definitions, we assessed diagnoses and medication separately and together. Second, to test potential severity of the exposure, we used various definitions of depression/anxiety including unplanned specialist visits (indicating seeking healthcare for acute symptoms) and records of diagnoses but without medication in the same period (indicating potentially untreated symptoms) as well as requiring cumulative exposure before, during and after pregnancy (indicating chronicity of symptoms). Third, to assess bias due to outcome misclassification, type 1 diabetes was based on either diagnosis or medication separately or requiring both. Last, in the main cohort born 2001–2019, we excluded all offspring that had no siblings and repeated the whole population analyses in order to evaluate the generalizability of the sibling comparison analysis. Significance levels were set at p<0.05. Data analysis was performed in Stata, V.17.0 (StataCorp LLC).

### Data and resource availability

The data used in this study are available from the respective sources outlined in the article, but restrictions apply and are therefore not publicly available. Requests can be made to the data providers after approval from the Swedish Ethical Review Authority. The principal investigator for this study may grant access to the pseudonymised data used on submission of a relevant research proposal and establishment of a data sharing agreement with Karolinska Institutet.

## Results

The cohort was composed of 1 807 809 mother-child pairs (online supplemental figure S1). In total, 113 068 (6.3%) offspring were exposed to maternal depression/anxiety *during pregnancy* and 200 220 (11.1 %) exposed any time *from before to after pregnancy*. Among those exposed *before pregnancy*, 70 475 (62.5%) continued being exposed *during pregnancy*, and 65 949 (55.6%) of those exposed *after pregnancy* had been exposed *during pregnancy* (online supplemental figure S3). Study individuals were followed for a mean 8.6 years (range 1 day to 19 years) from 1 year of age, with 8182 children (0.5%) developing type 1 diabetes at a mean age at onset of 7.9 years (SD 4.1). More mothers experiencing depression/anxiety *during pregnancy* had a history of type 1 diabetes (0.9% compared with 0.5%, table 1).

10.1136/bmjdrc-2023-003303.supp4 Supplementary data

**Table 1:** Descriptive statistics stratified by exposure to maternal depression/anxiety during pregnancy

	Overall (%) n=1 807 809	Exposed (%) n=1 13 068 (6.3)	Unexposed (%) n=1 694 741 (93.8)
Offspring characteristics			
Type 1 diabetes	8182 (0.5)	404 (0.4)	7778 (0.5)
Age at diagnosis, mean (SD), years	7.9 (4.1)	7.4 (4.0)	7.9 (4.1)
Sex			
Male	929 985 (51.4)	58 386 (51.6)	871 599 (51.4)
Birth year			
2002–2006	464 189 (25.7)	13 020 (11.5)	451 169 (26.6)
2007–2011	507 400 (28.1)	30 076 (26.6)	477 324 (28.2)
2012–2016	522 425 (28.9)	39 779 (35.2)	482 646 (28.5)
2017–2019	313 795 (17.4)	30 193 (26.7)	283 602 (16.7)
Maternal characteristics			
Early pregnancy body mass index, mean (SD), kg/m^2^	24.8 (4.7)	25.6 (5.3)	24.7 (4.6)
<18	22 974 (1.3)	1513 (1.3)	21 465 (1.3)
18–25	1 008 669 (55.8)	55 984 (49.5)	952 685 (56.2)
>25–30	422 230 (23.4)	28 670 (25.4)	393 560 (23.2)
>30	212 472 (11.8)	18 736 (16.6)	193 736 (11.4)
Missing	141 464 (7.8)	8165 (7.2)	133 299 (7.9)
Parity			
1	784 756 (43.4)	51 736 (45.8)	733 020 (43.3)
2	671 257 (37.1)	35 875 (31.7)	635 382 (37.5)
3	245 318 (13.6)	16 933 (15.0)	228 385 (13.5)
≥4	106 478 (5.9)	8524 (7.5)	97 954 (5.8)
Age at delivery, mean (SD), years	30.3 (5.1)	30.6 (5.4)	30.3 (5.1)
Type 1 diabetes	9513 (0.5)	966 (0.9)	8547 (0.5)
Highest level of educational attainment, years			
0–9	148 233 (8.2)	14 270 (12.6)	133 963 (7.9)
10–12	655 146 (36.2)	45 325 (40.1)	609 821 (36.0)
>12	991 319 (54.8)	52 971 (46.9)	938 348 (55.4)
Missing	13 111 (0.7)	502 (0.4)	12 609 (0.7)
History of asthma	195 089 (10.8)	20 925 (18.5)	174 164 (10.3)
Paternal characteristics			
Depression/anxiety during pregnancy	55 445 (3.1)	10 058 (8.9)	45 387 (2.7)

### Exposure to maternal depression/anxiety during pregnancy

**Figure 2:**
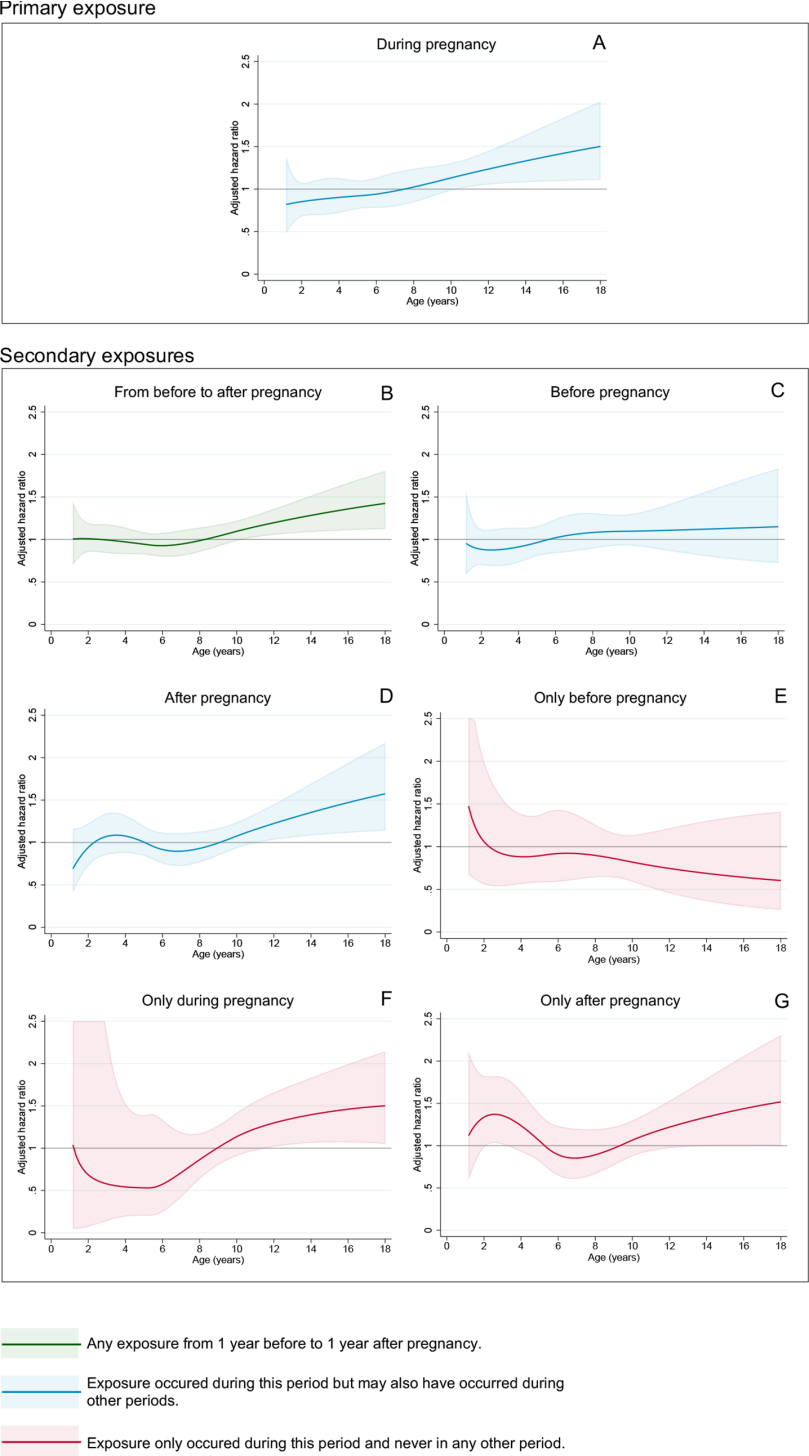
Association between maternal depression/anxiety during pregnancy and type 1 diabetes presented as time-varying HR of type 1 diabetes by attained age as well as timing-of-maternal-exposure comparisons across time periods before, during, and/or after pregnancy. (A) During pregnancy. (B) From before to after pregnancy. (C) Before pregnancy. (D) After pregnancy. (E) Only before pregnancy. (F) Only during pregnancy. (G) Only after pregnancy. (A) is the primary exposure. (B)–(G) are secondary exposures. All HRs with 95% CI are generated from flexible parametric models, adjusted for offspring birth year and sex, and maternal early pregnancy BMI, parity, age at delivery, type 1 diabetes, and highest level of educational attainment, additionally allowing for interaction between time and offspring birth year and sex.

**Figure 3:**
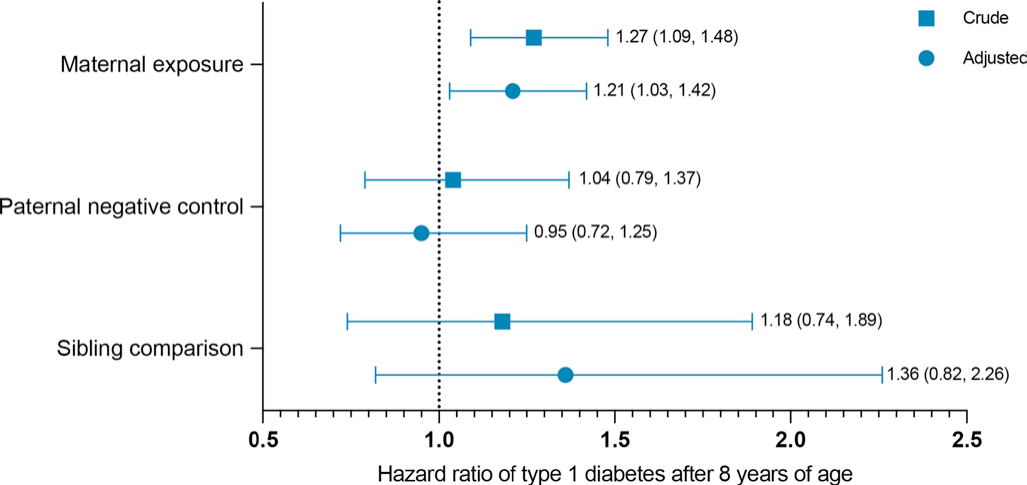
Association between maternal depression/anxiety during pregnancy and offspring type 1 diabetes after 8 years of age with paternal negative control and sibling comparison. HRs are presented with 95% CIs, crude and adjusted. Maternal exposure models were adjusted for offspring birth year and sex as well as maternal early pregnancy BMI, parity, age at delivery, type 1 diabetes, and highest level of educational attainment. Paternal negative control models were adjusted for offspring birth year and sex as well as paternal age at delivery, type 1 diabetes, and highest level of educational attainment. Sibling comparison models were adjusted for offspring birth year and sex as well as maternal early pregnancy BMI, parity, and age at delivery.

The association between the primary exposure maternal depression/anxiety *during pregnancy* and offspring type 1 diabetes is displayed in figure 2 with an increased risk starting at around 8 years of age (figure 2A). After adjustment, HRs were smaller but followed the same pattern as in the crude model (online supplemental figure S4). In both crude and adjusted Cox models, maternal depression/anxiety *during pregnancy* was associated with offspring type 1 diabetes after 8 years of age (adjusted (a)HR 1.21 (95% CI 1.03 to 1.42)) but not before 8 years of age (0.91 (0.78 to 1.04), figure 3, online supplemental table S1). We found no evidence of effect modification by maternal BMI, type 1 diabetes or asthma (p values of tests for interactions ranged from 0.13 to 0.62).

10.1136/bmjdrc-2023-003303.supp5 Supplementary data

### Timing-of-exposure comparisons

In secondary exposure periods, HR curves for the *from before to after pregnancy* period (figure 2B), for the *after pregnancy* period (figure 2D), as well as for the *only during pregnancy* (figure 2F) or *only after pregnancy* (figure 2G) periods had a similar shape to the primary exposure analysis with increasing HRs after 8 years of age. In contrast, for the *before pregnancy* (figure 2C) and *only before pregnancy* (figure 2E) periods, the HR curves between maternal depression/anxiety and type 1 diabetes did not show any changes of note. All estimates from the corresponding Cox models are presented in online supplemental table S1. For example, rates of type 1 diabetes were increased if exposure occurred *only during* (aHR 1.24 (95% CI 0.96 to 1.60)) or *only after pregnancy* (1.14 (0.91 to 1.44)), but were not for exposure *only before pregnancy* (0.91 (0.64 to 1.30)).

### Paternal negative control

Counter to maternal *during pregnancy* exposure, the association between fathers’ depression/anxiety *during pregnancy* and offspring type 1 diabetes after 8 years of age was close to null (figure 3, aHR 0.95 (95% CI 0.72 to 1.25)). Model estimates, and characteristics stratified on paternal exposure, are presented in online supplemental tables S2 and S3.

### Sibling comparison

When comparing offspring exposed to maternal depression/anxiety *during pregnancy* with their siblings unexposed to maternal depression/anxiety during their own gestation, the HR of type 1 diabetes after 8 years of age remained positive (aHR 1.36 (95% CI 0.82 to 2.26)) in relation to the whole population analysis, although with wide CIs including 1 (figure 3, online supplemental table S2).

### Sensitivity analyses

Maternal and offspring characteristics were similar in offspring born 2006–2019 (online supplemental table S4), although fewer children developed type 1 diabetes (N=3943 (0.3%)) due to shorter follow-up time (mean 6.6 years, range 1 day to 15 years). As in the primary analysis of the whole cohort, an increased rate of type 1 diabetes among those exposed to depression/anxiety *during pregnancy* was found in the restricted cohort with full register coverage (aHR of type 1 diabetes >8 years of age 1.16 (95% CI 0.95 to 1.43)). Assessing exposure separately for diagnosis or medication of maternal depression/anxiety yielded diminished results when based on diagnosis only (0.91 (0.62 to 1.33)) and commensurate results to the primary analysis when based on medication only (1.24 (1.00 to 1.53)). However, several of these alternative exposure definitions including those intending to capture acute, untreated, or chronic symptoms had few observations, reflected by large CIs (online supplemental table S5). Stricter outcome definitions for type 1 diabetes diagnosis or insulin prescription showed comparable results to the primary analysis (online supplemental table S6). Finally, results of analyses based on a subsample with only siblings were akin to whole population estimates (online supplemental table S7).

## Discussion

In this nationwide cohort of 1.8 million Swedish mother-child pairs, we demonstrate an association between exposure to maternal depression/anxiety during pregnancy and offspring development of type 1 diabetes after, but not before, 8 years of age. Timing-of-exposure comparisons indicate the importance of during and after pregnancy exposures. Additionally, the null result when using exposure to paternal depression/anxiety during pregnancy as a negative control, and rather unchanged estimates in the sibling comparison, support the conclusion that the demonstrated association is unlikely to be entirely confounded by shared familial factors.

This is the first study investigating maternal depression/anxiety during and around the pregnancy period as a risk factor for type 1 diabetes. Previous research on prenatal early-life stress has focused on alternative measures of stress during pregnancy such as bereavement and adverse life events. Similarly to our findings, a population-based Danish study by Virk *et al*^10^ reported an increased rate of type 1 diabetes in offspring after maternal exposure to death of a sibling or father during pregnancy (incidence rate ratio 1.23 (95% CI 1.18 to 1.64)) that was increased if the death was due to unnatural causes (2.03 (1.22 to 3.38)). On the other hand, smaller birth cohort studies found no or small overall associations between various severe adverse life events during pregnancy such as unemployment, violence, or divorce and offspring type 1 diabetes, even in genetically at-risk populations.^11–13^ Differences in the potential biological effects depending on the type, severity and timing of the stressor, the child’s genetic risk as well as sample size (potentially hindering uncovering age-varying or small effects) may explain the conflicting results.

In addition, we demonstrate age-dependent effects of maternal depression/anxiety during pregnancy, with the risk of type 1 diabetes only increased after, but not before, 8 years of age. This could be due to different risk factors and mechanisms associated with an earlier onset of disease within the first years of life compared with onset later on in childhood. For instance, early onset type 1 diabetes is more often associated with human leukocyte antigen-mediated genetic susceptibility,^28^ which is not linked with maternal stress during pregnancy, and might explain why we did not find an increased risk among younger children. This is in line with the growing body of current research on disease heterogeneity in type 1 diabetes and the concept of endotypes with different underlying disease pathways.^29^ Age-varying differences in risk factors for the progression from autoantibody positivity to clinical disease as well as in characteristics at diagnosis of type 1 diabetes have been shown.^30–32^

While other studies on early-life stress and type 1 diabetes have not explicitly differentiated between exposure during and around pregnancy, we attempted to understand differences depending on the timing of exposure. Exposure *before pregnancy* (a period where more than half of the women were also exposed *during pregnancy*) displayed a comparable association to the primary analysis *during pregnancy*, but exposure that occurred *only before pregnancy* (a period not including any exposure *during pregnancy*) was not associated. In contrast, slightly stronger associations were found when exposure occurred *only during pregnancy*, highlighting the specific importance of the pregnancy period. Associations between maternal depression/anxiety and offspring type 1 diabetes remained similar also in the secondary exposures including *after* or *only after pregnancy*. Although a large proportion of those exposed *after pregnancy* in our data had in fact been exposed *during pregnancy*, these exposures may moreover either represent women with symptoms during pregnancy that for a number of possible reasons did not medicate during pregnancy, or a different phenotype altogether such as postpartum depression. Our identification of *after pregnancy* exposures as predictors of offspring type 1 diabetes is consistent with several studies that have investigated various parental and child stress exposures during infancy.^6^ Since an exposure that occurs *only after pregnancy* cannot entail fetal programming, these findings do not contradict our main results of an association with exposure *during pregnancy*, but rather underscore the possibility of different pathways of etiopathogenesis.

To examine potential unmeasured familial confounding in the relationship between maternal depression/anxiety *during pregnancy* and offspring type 1 diabetes, we used both paternal negative control and sibling comparison. The null finding between fathers’ depression/anxiety *during pregnancy* and offspring type 1 diabetes, as well as the direction and magnitude of the estimates when comparing the whole population to the sibling comparison, does not suggest that shared environmental or genetic factors to a large extent explain our findings of an increased risk after 8 years of age. Familial coaggregation has been demonstrated between depression/anxiety and type 1 diabetes,^16^ although that partly may be attributed to causal effects. Furthermore, the influence of shared environmental factors to the coaggregation seems to be small and evidence for shared genetic influences has not been found.^33^

Even though we cannot fully rule out residual confounding, the association demonstrated may in fact represent a causal pathway. One possible mechanism is that stress during pregnancy could contribute to fetal programming and initiation of autoimmunity. Maternal stress has, via the hypothalamic-pituitary-axis, been shown to promote immune system dysregulation and drive proinflammatory processes.^34^ Another likelihood is that maternal stress during pregnancy impacts downstream maternal or offspring factors (environment-environment interplay) that in turn might increase the risk of or trigger diabetes progression, especially in already susceptible individuals (environment-gene interplay). For instance, maternal stress during pregnancy is associated with childhood asthma, infections, and obesity.^23 35 36^ In turn, these conditions are linked to an increased risk of subsequent type 1 diabetes.^22 37 38^ Alternatively, in the specific case of exposure to maternal depression/anxiety, the association with offspring type 1 diabetes could potentially be explained by either the stress of the illness itself or the medication used to treat the condition. Future research will be instrumental to help better understand these pathways.

### Strengths and limitations

Our study has several strengths. Importantly, this large, nationwide sample covers almost all births in Sweden over an 18-year long period with sufficient prospective follow-up to uncover age-varying associations. The results are consequently highly generalizable without selection or recall bias. The register-based nature of the study also enabled unequivocal linkage of multiple rich data sources, allowing for a life-course approach from preconception through gestation, infancy, and into childhood. We adjusted for a range of confounders, compared across exposure periods, and applied family-designs based on fathers and siblings to assess the impact of familial confounding. Moreover, basing the definition of type 1 diabetes on diagnoses ought to accurately have captured cases given that children are routinely hospitalized on diabetes onset, ICD-10 has specific codes for various forms of diabetes to avoid misclassification compared with historical ICD-versions, and other forms of diabetes under 18 years of age are rare.^39^ Although we cannot refute possible alternative indications for insulin therapy, using insulin prescription as an epidemiological definition for type 1 diabetes has been validated in Swedish material.^21^ Sensitivity analyses displayed robust results independent of the outcome definition used (diagnosis, insulin, or both).

Our findings should also be interpreted in light of several limitations. First, the NPR does not contain diagnoses of depression/anxiety from primary care which may have contributed to exposure misclassification. Fortunately, all dispensed medication prescriptions are included in the PDR, which allowed us to identify a large number of the women with a milder disease not requiring psychiatric specialist care. Prescription data capture the majority of all patients treated for depression (76%) or anxiety (63%) by general practitioners in Sweden.^40^ We will also have missed cases not seeking medical attention, not requiring, or for other reasons abstaining from medication during pregnancy. This bias ought to be non-differential in regard to the offspring’s type 1 diabetes and may have resulted in underestimation of a true association. In addition, combining a spectrum of diagnoses and medication enabled us to capture a proxy of stress, but we did not study differences between symptoms of depression compared with anxiety, or address actual treatment effects, as this was outside the scope of our research question.

Second, due to medication information registered in the PDR only from July 1, 2005 onward, exposure occurring during the first years of the cohort was to a higher extent based on diagnoses and may therefore represent a more severe phenotype of depression/anxiety. However, results of the sensitivity analysis in a restricted cohort born from July 1, 2006 with full register coverage displayed similar patterns as in the main analysis, indicating that this did not explain our findings.

Third, despite including almost 2 million children, because of the relatively rare outcome type 1 diabetes the study suffered from low statistical power in various sibling and subgroup analyses resulting in limited interpretations.

Finally, inherent limitations with sibling comparisons include amplification of potential residual confounding or of other biases in the main results.^41^ Finding similar estimates when repeating the main analysis in the sibling cohort does however speak to the generalizability of siblings to all children.

## Conclusion

In conclusion, maternal depression/anxiety specifically during pregnancy is associated with the onset of type 1 diabetes after 8 years of age. The triangulation of evidence in this study using several approaches including timing-of-exposure comparisons, paternal negative control, and sibling comparison sheds light on a potential causal pathway arising from fetal programming. These results emphasize the importance of the environmental early-life origins of type 1 diabetes. Continued research aiming to further understand the mechanisms through which stress during pregnancy, particularly related to symptoms, severity and treatment of maternal psychiatric illness, may contribute to the development of offspring type 1 diabetes, alongside replication of our findings in other settings, is warranted.

## Data Availability

Data are available on reasonable request. Data may be obtained from a third party and are not publicly available. The data used in this study are available from the respective sources outlined in the article, but restrictions apply and are therefore not publicly available. Requests can be made to the data providers after approval from the Swedish Ethical Review Authority. The principal investigator for this study may grant access to the pseudonymised data used on submission of a relevant research proposal and establishment of a data sharing agreement with Karolinska Institutet.
