# Supplementary material for: Maternal depression or anxiety during pregnancy and offspring type 1 diabetes: a population-based family-design cohort study
Source: BMJ Open Diabetes Res Care. 2023 Apr 20;11(2):e003303. doi: 10.1136/bmjdrc-2023-003303 (PMC10124198; doi:10.1136/bmjdrc-2023-003303)
Supplement: Supplementary data [file bmjdrc-2023-003303supp001.pdf]

Maternal depression or anxiety during pregnancy and offspring type 1 diabetes – a population-based family-design cohort study, Smew et al.

Supplemental Material – Table of contents

Supplemental Methods....2

Supplemental References....5

Supplemental Table S1. Associations between primary and secondary exposures of maternal depression/anxiety and offspring type 1 diabetes....7

Supplemental Table S2. Associations between maternal depression/anxiety during pregnancy and offspring type 1 diabetes with paternal negative control and sibling comparison....9

Supplemental Table S3. Descriptive statistics stratified on exposure to paternal depression/anxiety during pregnancy....10

Supplemental Table S4. Descriptive statistics stratified on exposure to maternal depression/anxiety during pregnancy in the restricted cohort born 2006–2019....11

Supplemental Table S5. Sensitivity analysis of the association between maternal depression/anxiety during pregnancy and offspring type 1 diabetes using alternative exposure definitions in the restricted cohort born 2006–2019....13

Supplemental Table S6. Sensitivity analysis of the association between maternal depression/anxiety during pregnancy and offspring type 1 diabetes using alternative outcome definitions in the restricted cohort born 2006–2019....15

Supplemental Table S7. Sensitivity analysis of the association between maternal depression/anxiety during pregnancy and offspring type 1 diabetes in subsamples of offspring with siblings from the whole population born 2001–2019....16

Supplemental Figure Legends....17

Supplemental methods

Register linkages

Data for this study were obtained through linkage of several nationwide population registers. These included, from Statistics Sweden, the Multi-generation register for linkage to parents and siblings (1); the Total Population Register for death and migration data; and the Longitudinal integrated database for labour market studies (Swedish acronym LISA) for information on parental educational attainments (2). From the National Board of Health and Welfare, our study population was identified from the Medical Birth Register (MBR) (3), including information on mother and child through pregnancy and delivery, and linked to the National Patient Register (NPR) that includes diagnoses from all specialist in-patient and approximately 80% of out-patient care since 2001, according to the International Classification of Diseases (ICD) (4); and the Prescribed Drug Register (PDR) containing all prescribed dispensations of medications according to the Anatomical Therapeutic Classification (ATC) starting 1 July 2005 (5). Data were available through 2021.

Covariates

Maternal and offspring related characteristics were obtained from the MBR and included body mass index (BMI, <18/18–25/>25–30/>30 kg/m^2) from the first antenatal care visit in gestational weeks 10–12, parity (1, 2, 3, ≥4 children), and age at delivery (continuous, in years), as well as offspring sex (male/female) and calendar year of birth.

Parental history of type 1 diabetes was based on the same definition as the offspring outcome, i.e., any diagnosis of type 1 diabetes (ICD-8 and 9: 250, ICD-10: E10) registered in the NPR or

any dispensation of insulin prescription (ATC A10A) in the PDR, both before 18 years of age (6,7). However, given that the oldest parents in the cohort (born 1987 or before) would not be able to have a first registration of insulin before 18 years of age in the PDR starting 2005, we required additional validated criteria to fulfill the definition based on insulin prescription: at least one prescription if male or three prescriptions if female (to avoid misclassification of gestational diabetes) and no prescription for any oral antidiabetic drugs ever for either (8).

The highest level of educational attainment (0–9/10–12/>12 years of school) for mothers and fathers was collected from LISA.

Maternal history of asthma was defined as at least one of the following: 1) diagnosis of asthma in the NPR before childbirth (ICD-8 and 9: 493, ICD-10: J45, J46), 2) at least two prescriptions of anti-asthmatic medication, including inhaled beta-2 agonists or corticosteroids, or oral leukotriene-receptor antagonists, from the PDR before childbirth (ATC R03AC, R03AK, R03BA, R03DC), 3) self-reported asthma in the MBR at the first antenatal visit (9–11).

Directed acyclic graph

The directed acyclic graph (Supplemental Fig. S2) produced using dagitty.net (12) displays the association between maternal depression/anxiety during pregnancy (exposure) and offspring type 1 diabetes (outcome) in relation to other covariates chosen based on literature review and medical knowledge (potential confounders, mediators or effect modifiers). Green lines represent causal pathways, whereas red lines represent potential biased pathways.

Proposed confounders (defined as origins of exposure and outcome, but not on the causal pathway between exposure and outcome, in red) include birth year, highest level of maternal education (as a proxy of socioeconomic status), maternal type 1 diabetes, and the pregnancy-related factors maternal BMI, age at delivery, and parity. These variables were adjusted for in the models, including sex which may be a predictor of the outcome. Unmeasured confounding from familial factors was accounted for in comparison of timing of exposure analyses as well as in paternal negative control and sibling comparison.

Perinatal characteristics such as birth weight, gestational age and mode of delivery potentially lie on the causal pathway between exposure and outcome as mediators (in blue) and were not adjusted for, since conditioning on such intermediate variables may open alternative biased pathways (13,14). Given the relationship between maternal depression/anxiety and asthma (15,16), as well as between offspring asthma and type 1 diabetes (6,17), we investigated potential effect modification of the relationship between exposure and outcome under the influence of maternal asthma alongside testing for interaction by maternal type 1 diabetes and early pregnancy BMI.

Supplemental References

1. Ekbom A. The Swedish Multi-generation Register. Methods Mol Biol. 2011;675:215–20.
2. Ludvigsson JF, Almqvist C, Bonamy AKKE, Ljung R, Michaëlsson K, Neovius M, et al. Registers of the Swedish total population and their use in medical research. Eur J Epidemiol. 2016;31(2):125–36.
3. Centre for Epidemiology. The Swedish Medical Birth Register - A summary of content and quality [Internet]. Stockholm; 2003. Available from: https://www.socialstyrelsen.se/en/
4. Ludvigsson JF, Andersson E, Ekbom A, Feychting M, Kim JL, Reuterwall C, et al. External review and validation of the Swedish national inpatient register. BMC Public Health. 2011;11(1):450.
5. Wallerstedt SM, Wettermark B, Hoffmann M. The First Decade with the Swedish Prescribed Drug Register – A Systematic Review of the Output in the Scientific Literature. Basic Clin Pharmacol Toxicol. 2016;119(5):464–9.
6. Smew AI, Lundholm C, Sävendahl L, Lichtenstein P, Almqvist C. Familial Coaggregation of Asthma and Type 1 Diabetes in Children. JAMA Netw Open. 2020;3(3):e200834.
7. Wernroth ML, Fall K, Svennblad B, Ludvigsson JF, Sjölander A, Almqvist C, et al. Early Childhood Antibiotic Treatment for Otitis Media and Other Respiratory Tract Infections Is Associated With Risk of Type 1 Diabetes: A Nationwide Register-Based Study With Sibling Analysis. Diabetes Care. 2020;43(5):991–9.
8. Rawshani A, Landin-Olsson M, Svensson AM, Nyström L, Arnqvist HJ, Bolinder J, et al. The incidence of diabetes among 0-34 year olds in Sweden: New data and better methods. Diabetologia. 2014;57(7):1375–81.
9. Brew BK, Lundholm C, Viktorin A, Lichtenstein P, Larsson H, Almqvist C. Longitudinal depression or anxiety in mothers and offspring asthma: a Swedish population-based study. Int J Epidemiol. 2018;47(1):166–174.
10. Rejnö G, Lundholm C, Larsson K, Larsson H, Lichtenstein P, D'Onofrio BM, et al. Adverse Pregnancy Outcomes in Asthmatic Women: A Population-Based Family Design Study. J Allergy Clin Immunol Pract. 2018;6(3):916-922.e6.
11. Rejnö G, Lundholm C, Gong T, Larsson K, Saltvedt S, Almqvist C. Asthma during Pregnancy in a Population-Based Study - Pregnancy Complications and Adverse Perinatal Outcomes. PLoS One. 2014;9(8):e104755.
12. Textor J, van der Zander B, Gilthorpe MS, Liśkiewicz M, Ellison GT. Robust causal inference using directed acyclic graphs: the R package "dagitty." Int J Epidemiol. 2016;45(6):1887–94.

13. Vanderweele TJ, Mumford SL, Schisterman EF. Conditioning on intermediates in perinatal epidemiology. Epidemiology. 2012;23(1):1–9.

14. Hernández-Díaz S, Schisterman EF, Hernán MA. The Birth Weight "Paradox" Uncovered? Am J Epidemiol. 2006;164(11):1115–20.

15. Rejnö G, Lundholm C, Öberg S, Lichtenstein P, Larsson H, D'Onofrio B, et al. Maternal anxiety, depression and asthma and adverse pregnancy outcomes – a population based study. Sci Rep. 2019;9(1):13101.

16. Tedner SG, Lundholm C, Olsson H, Almqvist C. Depression or anxiety in adult twins is associated with asthma diagnosis but not with offspring asthma. Clinical & Experimental Allergy. 2016;46(6):803–12.

17. Sgrazzutti L, Sansone F, Attanasi M, di Pillo S, Chiarelli F. Coaggregation of Asthma and Type 1 Diabetes in Children: A Narrative Review. Int J Mol Sci. 2021;22(11).

Supplemental Tables

Supplemental Table S1. Associations between primary and secondary exposures of maternal depression/anxiety before, during or/and after pregnancy and offspring type 1 diabetes, stratified by attained age (1–8, >8 years of age).

	No of cases (incidence rate per 10 000 person-years)				
	**Exposed**	**Unexposed**	**Crude HR (95% CI)**	**HR (95% CI)***	**HR (95% CI)**[†]
**Primary exposure**					
During pregnancy					
1–8 years of age	228 (4.13)	4171 (4.43)	0.95 (0.83, 1.08)	0.91 (0.79, 1.04)	0.91 (0.78, 1.04)
>8 years of age	176 (8.60)	3607 (6.73)	1.27 (1.09, 1.48)	1.21 (1.03, 1.42)	1.22 (1.04, 1.42)
**Secondary exposures**					
1 year before pregnancy to 1 year after delivery					
1–8 years of age	440 (4.43)	3959 (4.41)	1.02 (0.92, 1.12)	0.98 (0.88, 1.08)	0.98 (0.88, 1.08)
>8 years of age	301 (8.22)	3482 (6.70)	1.22 (1.08, 1.37)	1.17 (1.03, 1.32)	1.17 (1.03, 1.32)
Before pregnancy					
1–8 years of age	236 (4.40)	4163 (4.41)	1.02 (0.89, 1.16)	0.95 (0.83, 1.09)	0.95 (0.83, 1.09)
>8 years of age	131 (8.85)	3652 (6.74)	1.31 (1.10, 1.56)	1.16 (0.97, 1.40)	1.16 (0.97, 1.40)
After pregnancy					
1–8 years of age	254 (4.39)	4145 (4.41)	1.01 (0.89, 1.15)	0.97 (0.85, 1.11)	0.97 (0.85, 1.11)
>8 years of age	169 (8.36)	3614 (6.74)	1.23 (1.05, 1.43)	1.14 (0.97, 1.34)	1.14 (0.97, 1.34)
Only before pregnancy					
1–8 years of age	77 (4.51)	4322 (4.41)	1.04 (0.83, 1.30)	0.99 (0.78, 1.26)	0.99 (0.78, 1.26)
>8 years of age	34 (6.63)	3749 (6.80)	0.97 (0.69, 1.36)	0.91 (0.64, 1.30)	0.91 (0.64, 1.30)

Smew AI, *et al. BMJ Open Diab Res Care* 2023; 11:e003303. doi: 10.1136/bmjdrc-2023-003303

Only during pregnancy					
1–8 years of age	57 (3.97)	4342 (4.42)	0.90 (0.69, 1.17)	0.90 (0.69, 1.18)	0.90 (0.69, 1.18)
>8 years of age	66 (8.12)	3717 (6.78)	1.20 (0.94, 1.53)	1.24 (0.96, 1.60)	1.24 (0.96, 1.60)
Only after pregnancy					
1–8 years of age	115 (4.92)	4284 (4.40)	1.13 (0.94, 1.36)	1.13 (0.94, 1.37)	1.13 (0.94, 1.37)
>8 years of age	81 (8.15)	3702 (6.77)	1.18 (0.95, 1.48)	1.14 (0.91, 1.44)	1.14 (0.91, 1.44)

Footnote:
[*]Models were adjusted for offspring birth year and sex as well as maternal early pregnancy BMI, parity, age at delivery, type 1 diabetes and highest level of educational attainment.
[†]Models were additionally adjusted for paternal depression/anxiety.

Supplemental Table S2. Associations between maternal depression/anxiety during pregnancy and offspring type 1 diabetes after 8 years of age with paternal negative control and sibling comparison.

	No of cases (incidence rate per 10 000 person-years)				
	**Exposed**	**Unexposed**	**Crude HR (95% CI)**	**HR (95% CI)***	**HR (95% CI)†**
Maternal exposure	176 (8.60)	3607 (6.73)	1.27 (1.09, 1.48)	1.21 (1.03, 1.42)	1.21 (1.03, 1.42)
Paternal negative control	52 (7.17)	3731 (6.79)	1.04 (0.79, 1.37)	0.96 (0.73, 1.26)	0.95 (0.72, 1.25)
Sibling comparison‡	93 (7.97)	2410 (6.74)	1.18 (0.74, 1.89)	1.37 (0.83, 2.28)	1.36 (0.82, 2.26)

Footnote:
*Maternal exposure models were adjusted for offspring birth year and sex as well as maternal early pregnancy BMI, parity, age at delivery, type 1 diabetes and highest level of educational attainment. Paternal negative control models were adjusted for offspring birth year and sex as well paternal age at delivery, type 1 diabetes and highest level of educational attainment. Sibling comparison models were adjusted for offspring birth year and sex as well as maternal early pregnancy BMI, parity and age at delivery.
†Whole population and sibling comparison models were additionally adjusted for paternal depression/anxiety during pregnancy. Paternal negative control models were additionally adjusted for maternal depression/anxiety during pregnancy.
‡In sibling comparison models, only exposure and outcome discordant sibling pairs (N=297) were informative and contributed to the effect estimates. However, all siblings (N=1 265 116) were included in the calculation of incidence rates.

Supplemental Table S3. Descriptive statistics stratified on exposure to paternal depression/anxiety during pregnancy.

	**Overall (%)** n=1 807 809	**Exposed (%)** n=55 445 (3.1)	**Unexposed (%)** n=1 752 364 (96.9)
Child characteristics			
Type 1 diabetes	8182 (0.5)	165 (0.3)	8017 (0.5)
Age at diagnosis, mean (SD), years	7.9 (4.1)	6.2 (3.8)	7.9 (4.1)
Sex			
Male	929 985 (51.4)	28 480 (51.4)	901 505 (51.5)
Birth year			
2002–2006	464 189 (25.7)	3909 (7.1)	460 280 (26.3)
2007–2011	507 400 (28.1)	13 025 (23.5)	494 375 (28.2)
2012–2016	522 425 (28.9)	19 850 (35.8)	502 575 (28.7)
2017–2019	313 795 (17.4)	18 661 (33.7)	295 134 (16.8)
Paternal characteristics			
Age at delivery, mean (SD), years	33.7 (6.2)	34.7 (6.9)	33.6 (6.2)
Type 1 diabetes	9564 (0.5)	539 (1.0)	9025 (0.5)
Highest level of educational attainment, years			
0–9	191 176 (10.6)	9928 (17.9)	181 248 (10.3)
10–12	829 180 (45.9)	25 463 (46.0)	803 717 (45.9)
>12	737 683 (40.8)	19 768 (35.7)	717 915 (41.0)
Missing	49 770 (2.8)	286 (0.5)	49 484 (2.8)
Maternal characteristics			
Depression/anxiety during pregnancy	113 068 (6.3)	10 058 (18.1)	103 010 (5.9)

Smew AI, *et al. BMJ Open Diab Res Care* 2023; 11:e003303. doi: 10.1136/bmjdrc-2023-003303

Supplemental Table S4. Descriptive statistics stratified on exposure to maternal depression/anxiety during pregnancy in the restricted cohort born 2006–2019.

	**Overall (%)** n=1 390 395	**Exposed (%)** n=102 607 (7.4)	**Unexposed (%)** n=1 287 788 (92.6)
Child characteristics			
Type 1 diabetes	4741 (0.3)	302 (0.3)	4439 (0.3)
Age at diagnosis, mean (SD), years	6.5 (3.5)	6.7 (3.4)	6.5 (3.5)
Sex			
Male	715 397 (51.5)	52 866 (51.5)	662 531 (51.5)
Birth year			
2006–2009	345 918 (24.9)	19 515 (19.0)	326 403 (25.4)
2010–2013	414 036 (29.8)	27 301 (26.6)	386 735 (30.3)
2014–2017	421 195 (30.3)	35 294 (34.4)	385 901 (30.0)
2018–2019	209 246 (15.1)	20 497 (20.0)	188 749 (14.7)
Maternal characteristics			
Early pregnancy body mass index, mean (SD), kg/m^2	24.8 (4.7)	25.6 (5.3)	24.8 (4.7)
<18	18 322 (1.3)	1383 (1.4)	16 939 (1.3)
18–25	778 774 (56.0)	50 888 (49.6)	727 886 (56.5)
>25–30	331 429 (23.8)	26 140 (25.5)	305 289 (23.7)
>30	172 221 (12.4)	17 214 (16.8)	154 997 (12.0)
Missing	89 659 (6.5)	6982 (6.8)	82 677 (6.4)
Parity			
1	600 486 (43.2)	46 891 (45.7)	553 595 (43.0)
2	518 313 (37.3)	32 840 (32.0)	485 473 (37.7)
3	189 350 (13.6)	15 338 (15.0)	174 012 (13.5)
≥4	82 246 (5.9)	7538 (7.4)	74 708 (5.8)
Age at delivery, mean (SD), years	30.4 (5.1)	30.6 (5.4)	30.4 (5.1)
Type 1 diabetes	8221 (0.6)	916 (0.9)	7305 (0.6)
Highest level of educational attainment, years			
0–9	119 041 (8.6)	12 958 (12.6)	106 083 (8.2)
10–12	487 517 (35.1)	40 620 (39.6)	446 897 (34.7)

>12	771 689 (55.5)	48 555 (47.3)	723 134 (56.2)
Missing	12 148 (0.9)	474 (0.5)	11 684 (0.9)
History of asthma	161 587 (11.6)	19 562 (19.1)	142 025 (11.0)
Paternal characteristics			
Depression/anxiety during pregnancy	52 561 (3.8)	9786 (9.5)	42 775 (3.3)

Smew AI, *et al. BMJ Open Diab Res Care* 2023; 11:e003303. doi: 10.1136/bmjdrc-2023-003303

Supplemental Table S5. Sensitivity analysis of the association between maternal depression/anxiety during pregnancy and offspring type 1 diabetes, stratified by attained age (1–8, >8 years of age), and using alternative exposure definitions based on various diagnosis and medication combinations in the restricted cohort born 2006–2019.

	No of cases (incidence rate per 10 000 person-years)				
	**Exposed**	**Unexposed**	**Crude HR (95% CI)**	**HR (95% CI)***	**HR (95% CI)†**
**Alternative definitions of exposure during pregnancy**					
Diagnosis‡ or medication§					
1–8 years of age	192 (4.01)	2950 (4.46)	0.91 (0.78, 1.05)	0.87 (0.75, 1.01)	0.87 (0.75, 1.01)
>8 years of age	110 (9.28)	1489 (7.71)	1.21 (1.00, 1.47)	1.16 (0.95, 1.43)	1.16 (0.95, 1.43)
Any diagnosis					
1–8 years of age	67 (3.77)	3075 (4.45)	0.86 (0.67, 1.09)	0.79 (0.61, 1.03)	0.79 (0.61, 1.03)
>8 years of age	28 (6.85)	1571 (7.82)	0.89 (0.61, 1.29)	0.91 (0.62, 1.33)	0.91 (0.62, 1.33)
Any medication					
1–8 years of age	167 (4.05)	2975 (4.46)	0.92 (0.79, 1.07)	0.88 (0.75, 1.03)	0.88 (0.75, 1.04)
>8 years of age	103 (9.96)	1496 (7.68)	1.31 (1.07, 1.59)	1.24 (1.00, 1.53)	1.24 (1.00, 1.53)
Both diagnosis and medication					
1–8 years of age	42 (3.79)	3100 (4.44)	0.86 (0.64, 1.17)	0.78 (0.56, 1.08)	0.78 (0.56, 1.08)
>8 years of age	21 (8.19)	1578 (7.79)	1.07 (0.69, 1.64)	1.06 (0.68, 1.65)	1.06 (0.68, 1.65)

Smew AI, *et al. BMJ Open Diab Res Care* 2023; 11:e003303. doi: 10.1136/bmjdrc-2023-003303

Any diagnosis for unplanned visit					
1–8 years of age	21 (3.04)	3121 (4.45)	0.69 (0.45, 1.06)	0.62 (0.39, 0.99)	0.62 (0.39, 0.99)
>8 years of age	15 (9.78)	1584 (7.78)	1.28 (0.77, 2.12)	1.38 (0.83, 2.29)	1.38 (0.83, 2.29)
Any diagnosis, no medication					
1–8 years of age	25 (3.73)	3117 (4.44)	0.85 (0.57, 1.26)	0.82 (0.54, 1.24)	0.82 (0.54, 1.24)
>8 years of age	7 (4.60)	1592 (7.82)	0.60 (0.28, 1.25)	0.65 (0.31, 1.37)	0.65 (0.31, 1.37)
Additional diagnosis or medication both before and after pregnancy					
1–8 years of age	89 (3.87)	3056 (4.45)	0.88 (0.71, 1.09)	0.84 (0.68, 1.05)	0.84 (0.68, 1.05)
>8 years of age	51 (10.2)	1548 (7.74)	1.35 (1.02, 1.79)	1.16 (0.85, 1.57)	1.16 (0.85, 1.57)

Footnote:
*Models were adjusted for offspring birth year and sex as well as maternal early pregnancy BMI, parity, age at delivery, type 1 diabetes and highest level of educational attainment.
†Models were additionally adjusted for paternal depression/anxiety during pregnancy.
‡Diagnosis refers to diagnosis of or unplanned visit for any mood- or anxiety-related disorders (ICD-10 F30-34, F38, F39) in the National Patient Register.
§Medication refers to anxiolytic or antidepressant medication (ATC N05B, N06A) in the Swedish Prescribed Drug Register or Medical Birth Register.

Supplemental Table S6. Sensitivity analysis of the association between maternal depression/anxiety during pregnancy and offspring type 1 diabetes, stratified by attained age (1–8, >8 years of age), and using alternative outcome definitions in the restricted cohort born 2006–2019.

	No of cases (incidence rate per 10 000 person-years				
	**Exposed**	**Unexposed**	**Crude HR (95% CI)**	**HR (95% CI)***	**HR (95% CI)†**
**Alternative outcome definitions**					
Any type 1 diabetes diagnosis					
1–8 years of age	166 (3.47)	2682 (4.06)	0.86 (0.74, 1.01)	0.85 (0.72, 1.00)	0.85 (0.72, 1.00)
>8 years of age	87 (7.34)	1176 (6.09)	1.21 (0.97, 1.50)	1.18 (0.93, 1.48)	1.17 (0.93, 1.48)
Any insulin prescription					
1–8 years of age	183 (3.82)	2835 (4.29)	0.90 (0.78, 1.05)	0.86 (0.74, 1.01)	0.86 (0.74, 1.01)
>8 years of age	108 (9.11)	1446 (7.48)	1.23 (1.01, 1.49)	1.18 (0.96, 1.45)	1.18 (0.96, 1.45)
Both type 1 diabetes diagnosis and insulin prescription					
1–8 years of age	157 (3.28)	2567 (3.88)	0.85 (0.73, 1.00)	0.84 (0.71, 0.99)	0.84 (0.71, 0.99)
>8 years of age	85 (7.17)	1133 (5.86)	1.23 (0.98, 1.53)	1.20 (0.95, 1.51)	1.19 (0.95, 1.51)

Footnote:
*Models were adjusted for offspring birth year and sex as well as maternal early pregnancy BMI, parity, age at delivery, type 1 diabetes and highest level of educational attainment.
†Models were additionally adjusted for paternal depression/anxiety during pregnancy.

Supplemental Table S7. Sensitivity analysis of the association between maternal depression/anxiety during pregnancy and offspring type 1 diabetes stratified by attained age (1–8, >8 years of age), in subsamples of offspring with siblings from the whole population born 2001–2019.

	No of cases (incidence rate per 10 000 person-years)				
	**Exposed**	**Unexposed**	**Crude HR (95% CI)**	**HR (95% CI)***	**HR (95% CI)†**
**Sibling subsample** N=1 265 116 *During pregnancy*					
1–8 years of age	142 (4.13)	2008 (4.35)	0.96 (0.82, 1.14)	0.92 (0.78, 1.10)	0.92 (0.78, 1.10)
>8 years of age	93 (7.97)	2410 (6.74)	1.18 (0.96, 1.46)	1.11 (0.89, 1.38)	1.11 (0.89, 1.38)

Footnote:
*Models were adjusted for offspring birth year and sex as well as maternal early pregnancy BMI, parity, age at delivery, type 1 diabetes and highest level of educational attainment.
†Models were additionally adjusted for paternal depression/anxiety during pregnancy.

Supplemental Figure Legends

Supplemental Figure S1. Flowchart of selection of study participants from the general population based on multiple Swedish national data sources.

Supplemental Figure S2. Directed acyclic graph of the association between maternal depression/anxiety during pregnancy and offspring type 1 diabetes.

Supplemental Figure S3. Schematic overview of the number of exposed offspring to maternal depression/anxiety before, during, and/or after pregnancy. All percentages are based on the total number of offspring in the cohort.

Supplemental Figure S4. Association between maternal depression/anxiety during pregnancy and type 1 diabetes presented as time-varying hazard ratios of type 1 diabetes by attained age. Hazard ratios alongside 95% confidence intervals are generated from flexible parametric models. They are presented crude (left) and adjusted (right) for offspring birth year and sex, and maternal early pregnancy BMI, parity, age at delivery, type 1 diabetes and highest level of educational attainment, allowing for interaction between time and offspring birth year and sex.
