# Supplementary material for: Maternal depression or anxiety during pregnancy and offspring type 1 diabetes: a population-based family-design cohort study
Source: BMJ Open Diabetes Res Care. 2023 Apr 20;11(2):e003303. doi: 10.1136/bmjdrc-2023-003303 (PMC10124198; doi:10.1136/bmjdrc-2023-003303)
Supplement: Supplementary data [file bmjdrc-2023-003303supp003.pdf]

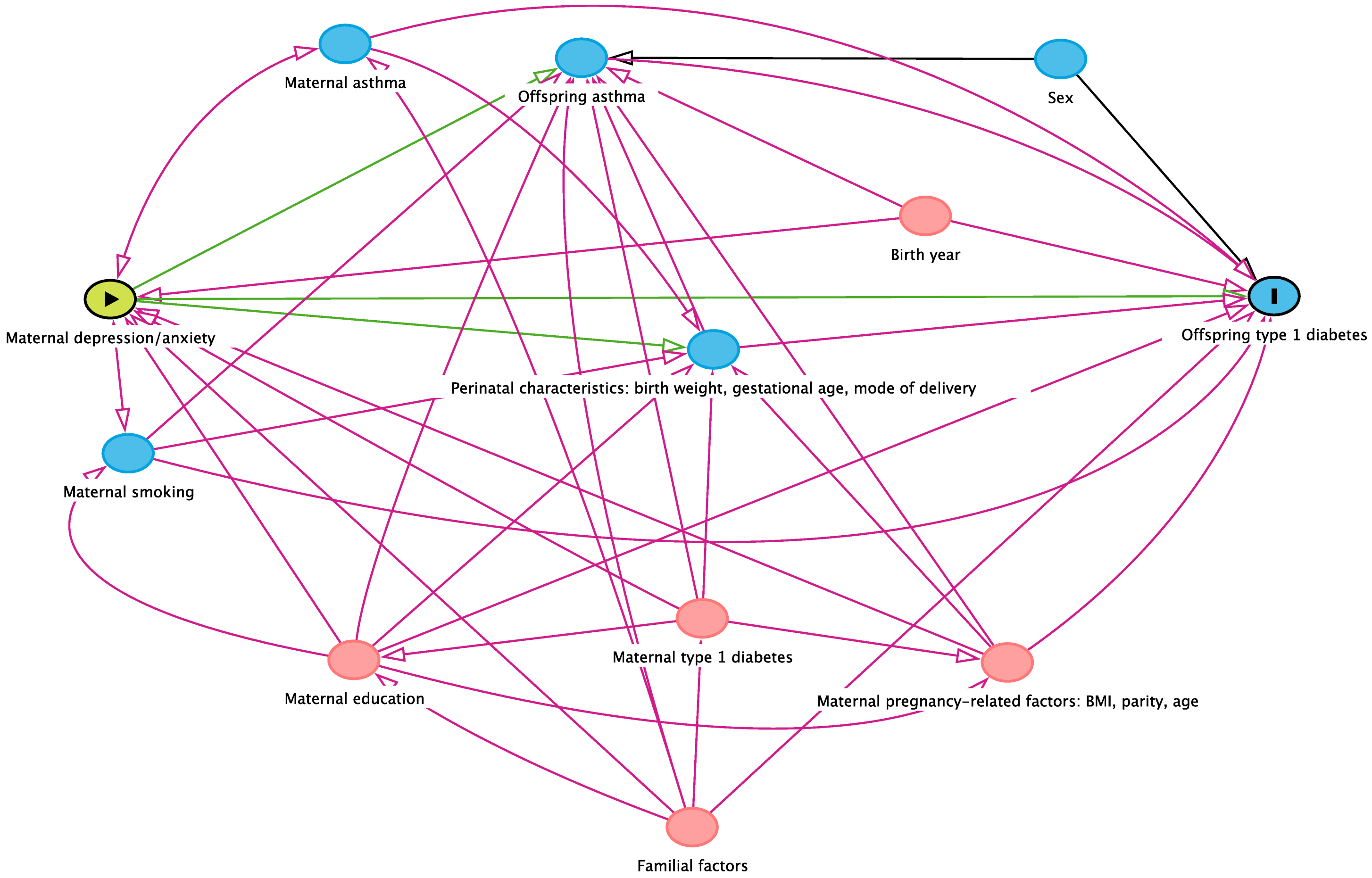

Supplemental Figure S2. Directed acyclic graph of the association between maternal depression/anxiety during pregnancy and offspring type 1 diabetes.
