# Supplementary material for: Maternal depression or anxiety during pregnancy and offspring type 1 diabetes: a population-based family-design cohort study
Source: BMJ Open Diabetes Res Care. 2023 Apr 20;11(2):e003303. doi: 10.1136/bmjdrc-2023-003303 (PMC10124198; doi:10.1136/bmjdrc-2023-003303)
Supplement: Supplementary data [file bmjdrc-2023-003303supp004.pdf]

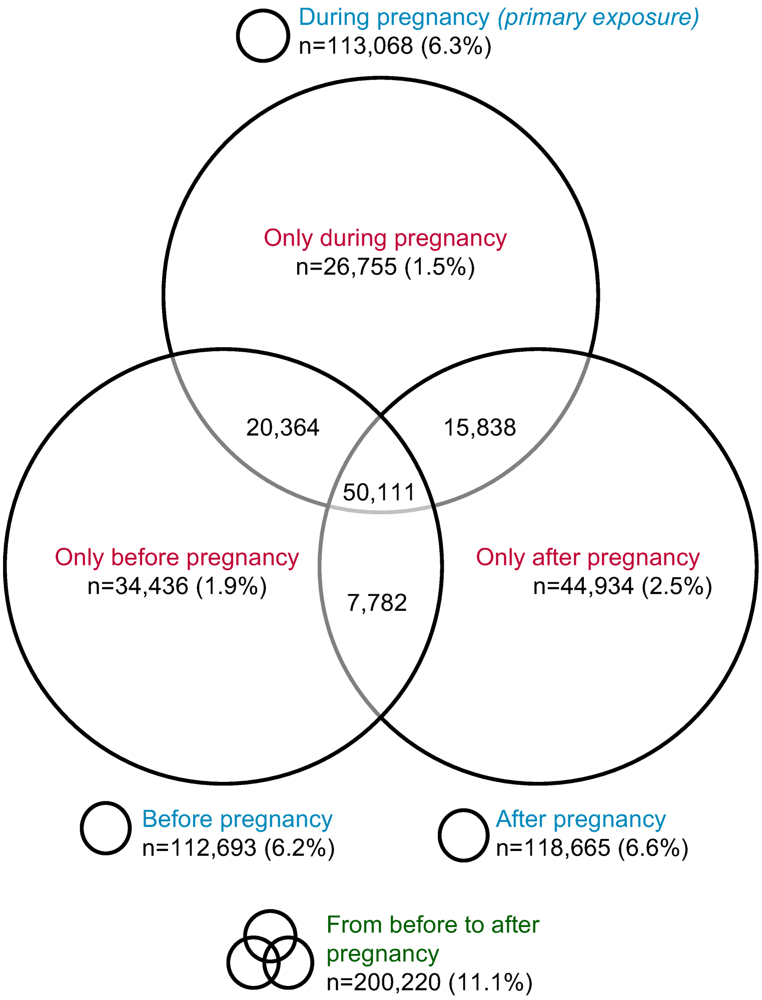

Green text — Any exposure from 1 year before to 1 year after pregnancy.

Blue text — Exposure occured during this period but may also have occurred during other periods.

Red text — Exposure only occured during this period and never in any other period.

Supplemental Figure S3. Schematic overview of the number of exposed offspring to maternal depression/anxiety before, during, and/or after pregnancy. All percentages are based on the total number of offspring in the cohort.
