# Supplementary material for: Maternal depression or anxiety during pregnancy and offspring type 1 diabetes: a population-based family-design cohort study
Source: BMJ Open Diabetes Res Care. 2023 Apr 20;11(2):e003303. doi: 10.1136/bmjdrc-2023-003303 (PMC10124198; doi:10.1136/bmjdrc-2023-003303)
Supplement: Supplementary data [file bmjdrc-2023-003303supp005.pdf]

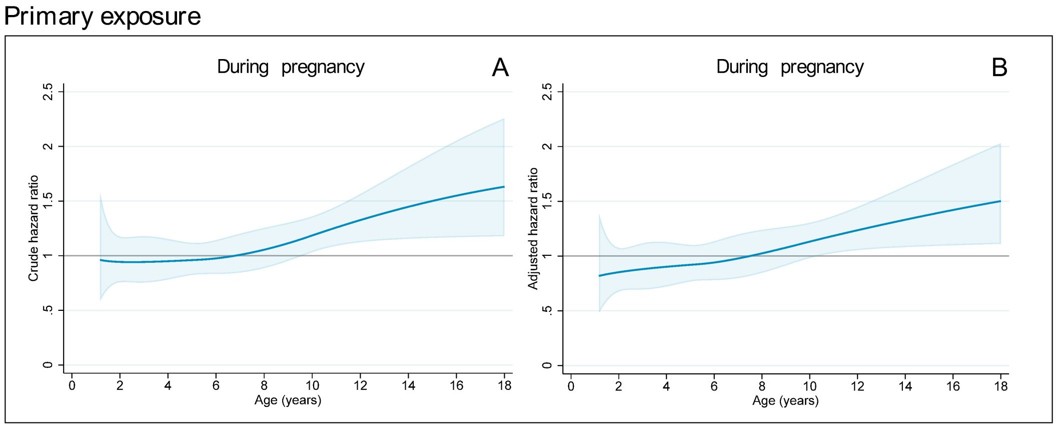

Supplemental Figure S4. Association between maternal depression/anxiety during pregnancy and type 1 diabetes presented as time-varying hazard ratios of type 1 diabetes by attained age. Hazard ratios alongside 95% confidence intervals are generated from flexible parametric models. They are presented crude (left) and adjusted (right) for offspring birth year and sex, and maternal early pregnancy BMI, parity, age at delivery, type 1 diabetes and highest level of educational attainment, allowing for interaction between time and offspring birth year and sex.
